# Investigation of contrast visual acuity with rigid gas permeable contact lenses after penetrating Keratoplasty

**DOI:** 10.1186/s12886-023-02769-9

**Published:** 2023-01-09

**Authors:** Ju Zhang, Xiao Lin, Xinhai Wang, Zhiwei Cheng, Xiaoxiao Li, Jicang He, Weiyun Shi, Hua Gao

**Affiliations:** 1 Shandong First Medical University ＆ Shandong Academy of Medical Sciences, Shandong Jinan, China; 2 Medical School of Ophthalmology and optometry, Shandong University of Traditional Chinese medicine, Jinan, Shandong China; 3 State Key Laboratory Cultivation Base, Shandong Provincial Key Laboratory of Ophthalmology, Shandong Eye Hospital, Shandong Eye Institute of Shandong First Medical University, 372 Jingsi Road, 250021 Jinan, Shandong China; 4 New England College of Optometry, Boston, MA USA

**Keywords:** Penetrating keratoplasty, Rigid gas permeable corneal contact lenses, Contrast visual acuity, Wavefront aberrations, Low contrast loss

## Abstract

**Background:**

To investigate the effects of rigid gas permeable contact lens (RGP-CL) wear on contrast visual acuity in patients after penetrating keratoplasty.

**Methods:**

Nineteen patients (19 eyes), aged 30.45 ± 5.83 years, who had received penetrating keratoplasty and were successfully fitted with RGP-CLs at our hospital from July 2017 to June 2018 were included. Contrast visual acuities at 100%, 25%, and 10% with spectacles and RGP-CLs were analyzed using the Chi-square test. The wavefront aberrations at the anterior surface of the cornea before and 1 month after RGP-CL wear were compared using the matched sample t-test.

**Results:**

The mean best spectacle-corrected visual acuities were 0.390 ± 0.135 logMAR, 0.706 ± 0.182 logMAR, and 0.952 ± 0.223 logMAR at the 100%, 25%, and 10% contrast levels, respectively, which were significantly lower than the RGP-CL-corrected visions at the three levels (0.255 ± 0.133 logMAR, 0.488 ± 0.168 logMAR, and 0.737 ± 0.159 logMAR; all *P* < 0.001). The vision losses with RGP-CLs were 0.231 ± 0.099 logMAR and 0.466 ± 0.094 logMAR at the 25% and 10% contrast levels, respectively. The Zernike spherical aberration Z^0^_4_ was reduced from 3.734 ± 1.061 μm to 2.622 ± 0.725 μm after wearing the RGP-CLs (*P* ≤ 0.001). The astigmatism parameters of Z^− 2^_2_ and Z^2^_2_ were also reduced from 3.761 ± 2.309 μm and 3.316 ± 2.147 μm to 2.637 ± 1.722 μm and 2.016 ± 1.184 μm, respectively (*P* < 0.05).

**Conclusion:**

For post-keratoplasty patients, RGP-CLs can help to improve visual performance, especially low contrast visual acuity. The improvement may be related to the reduction of corneal aberrations, mainly the spherical and astigmatism aberrations.

## Background

Corneal transplantation is the main approach to restoring the sight of patients with corneal blindness, [1–3] but the surgery usually induces irregularity of the front corneal surface, leading to poor postoperative visual acuity despite the correction with spectacles [4–6]. Although the implanted corneal graft becomes transparent, the poor visual performance may still affect the quality of life [7–9]. Rigid gas permeable contact lenses (RGP-CLs) have been reported to significantly improve visual acuity of patients undergoing keratoplasty for high permeability, safety, and correction of corneal astigmatism [7, 10, 11]. However, attention has just been paid to the vision under high contrast conditions. The reduced contrasts, which are more closely related to our daily activities, [12] have not been investigated. For keratoconic patients, wearing RGP-CLs could reduce second-order and higher-order aberrations in the cornea, and thereby improving the optical quality [13, 14]. But it is unclear whether different contrast visions could be increased for patients after keratoplasty by wearing the RGP-CLs, and whether there is a link to the reduced corneal aberrations. In this study, the visual and optical effects of RGP-CL wear on post-keratoplasty patients were evaluated by measuring contrast visual acuities (CVAs) at high, medium, and low levels and identifying the key corneal aberration change after lens wear, so as to provide an individualized further intervention for each patient with prior treatment of keratoplasty.

## Subjects and methods

### Patients

Nineteen post-keratoplasty patients (19 eyes) who were successfully fitted with RGP-CLs at our hospital from July 2017 to June 2018 were included. There were 14 males and 5 females, aged 16–48 years (mean, 30.45 ± 5.83 years). All of them had the sutures of penetrating keratoplasty removed at least 3 months before, with best spectacle-corrected visual acuity > 0.3 logMAR and corneal endothelial cell count ≥ 1000/mm^2^. They all expected better visual acuity, accepted RGP-CL wearing, and were not allergic to RGP-CL solutions. Patients who suffered active conjunctivitis or keratitis, allergic conjunctivitis, moderate or severe dry eye were excluded. Informed consent was obtained from all subjects or their guardians after the nature and possible consequences of the study had been explained to them. The study had an approval from the Shandong Eye Hospital Ethics Committee (SDSYKYY20170521) and complied with the tenets of the Declaration of Helsinki.

The primary diseases included keratoconus in 17 eyes, herpes simplex keratitis in 1 eye and fungal keratitis in 1 eye. The average time between initiation of RGP-CL wear and keratoplasty was 4.4 ± 2.0 years. Data of CVAs with spectacles and RGP-CLs as well as the wavefront aberrations at the anterior surface of the cornea before and 1 month after continuous wearing of RGP-CLs were collected. Before wearing RGP-CLs, corneal topography (Allegro Oculyzer, WaveLight GmbH, Erlangen, Germany) revealed significant irregular astigmatism with an index surface variance of 108.91 ± 46.26 and an index height asymmetry of 28.57 ± 18.73 [15]. The mean subjective spherical diameter was − 3.41 ± 3.50 D, and the column diameter was − 4.33 ± 2.02 D.

### Measurement of contrast visual acuity

The included eyes were tested for the uncorrected visual acuity (UCVA), best spectacle-corrected visual acuity (BSCVA), and RGP-CL-corrected visual acuity at 5.5 m from a multifunctional visual acuity tester (MFVA-100, BriteEye Medical Tech Co. Ltd, Shenzhen, China), which is capable of measuring distant visual acuity using isolate letters at four contrast levels (100%, 25%, 10%, and 5%), [16] in a dark room, with the contralateral eyes completely covered. Data at the levels of 100%, 25% and 10% were statistically analyzed, since the UCVA at 5% was over 1.4 logMAR after keratoplasty, exceeding the measurement range of the instrument. Low contrast loss was defined as low contrast visual acuity minus high contrast visual acuity [17, 18].

### Measurement of corneal wavefront aberrations

The wavefront aberrations of the anterior corneal surface before and 1 month after RGP-CL wear were examined in all included eyes. After the patient was acclimated to the dark room for 10 min, corneal topography was performed to measure the corneal surface in a naturally dilated state, 3–5 times for each eye. Three measurements with a good repeatability of 6 mm or more were selected to calculate the corneal aberrations in Zernike polynomials. The RMS values of the anterior surface aberrations in the central 8-mm area of the cornea, including spherical aberration Z_4_^0^, vertical coma aberration Z_3_ ^− 1^, horizontal coma aberration Z_3_ ^+ 1^, astigmatism term Z_2_ ^− 2^, and Z_2_^2^, were recorded in microns (µm) for statistical analysis.

### RGP-CL fitting

**Fig. 1:**
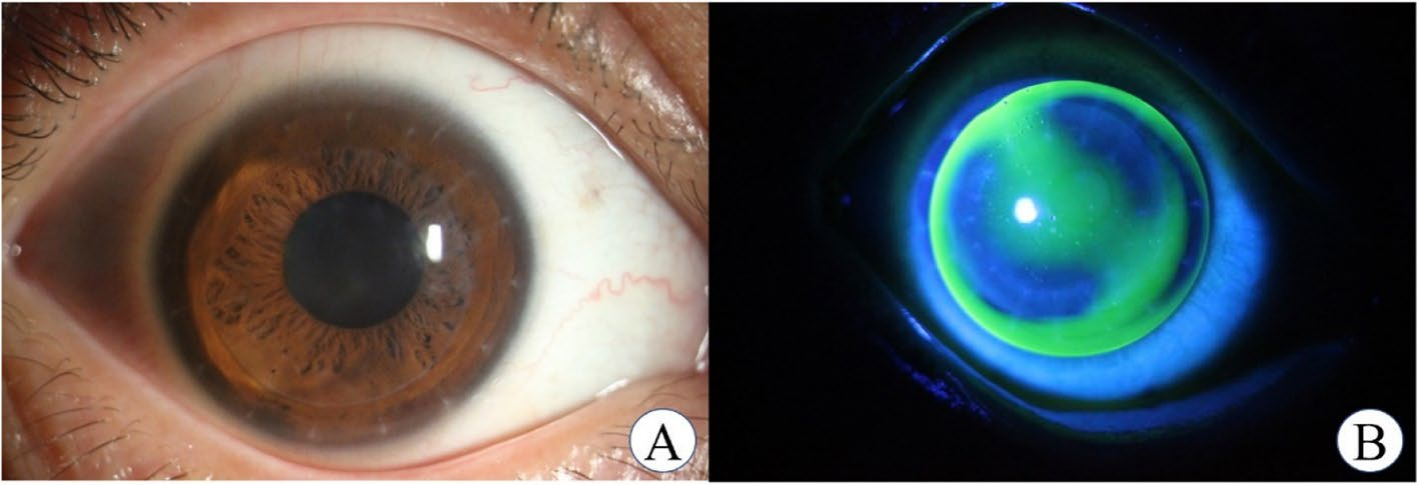
**A** The graft is transparent with all the sutures removed after keratoplasty. **B** The RGP-CL fitting is good for the eye with the thickness of the central lacrimal layer being 0.1 to 0.2 mm and the peripheral curve being 0.6 mm

FreshKon® Rose-K postoperative irregular lenses with a diameter of 10.0 to 10.6 mm were used in the study. The lens material is Boston XO, giving a nominal oxygen transmissibility of 100 × 10^− 11^(cm^2^/s) (mlO_2_/mL·mmHg). The RGP-CL was prepared according to the corneal parameters of each patient. The eye covered with the RGP-CL was stained with sodium fluorescein for observation of the distribution of tear fluids under the lens in a cobalt blue light of a slit lamp microscope. The fitting status was assessed and photographed. With staining, a tear fluid accumulation of 0.1 to 0.2 mm in the central cornea and a peripheral curve of 0.6 to 1.0 mm were detected. All lenses were well fitted with no obvious deviation, despite 1- to 2-mm movement (shown in Fig. 1).

### Statistical analysis

SPSS 19.0 software was used for statistical analysis. The data followed a normal distribution through the Kolmogorov-Smirnov test. Different corrective modalities under varied contrast visual acuity conditions were compared using the Chi-square test, and the data before and after RGP-CL wearing was analyzed using the matched sample t-test. *P* < 0.05 was considered statistically significant.

## Results

### UCVA, BSCVA, and RGP-CL-corrected visual acuity at different contrast levels

At the 100% contrast, UCVA was below 0.3 logMAR in all patients, BSCVA was above 0.3 logMAR in 21.1% (4/19) of the patients, and RGP-CL-corrected vision was above 0.3 logMAR in 63.2% (12/19) of the patients. The UCVA, BSCVA, and RGP-CL-corrected visual acuity at the 100%, 25%, and 10% contrasts were found to be statistically significant (*P* < 0.001) (shown in Table 1).

**Table 1:** Comparison of UCVA, BSCVA, and RGP-CL-corrected visual acuity at different contrast levels

Contrast visual acuity	UCVA	BSCVA	RGP-CL-corrected visual acuity	χ^2^	*P*
100%	0.801 ± 0.215	0.390 ± 0.135	0.255 ± 0.133	31.679	0.000
25%	1.181 ± 0.266	0.706 ± 0.182	0.488 ± 0.168	27.908	0.000
10%	1.363 ± 0.062	0.952 ± 0.223	0.737 ± 0.159	32.990	0.000
Data are presented as mean ± standard deviation. *P* values stand for comparison of different groups. *UCVA * uncorrected visual acuity, *BSCVA * best spectacle corrected visual acuity					

### The difference between BSCVA and RGP-CL-corrected visual acuity at different contrast levels

**Fig. 2:**
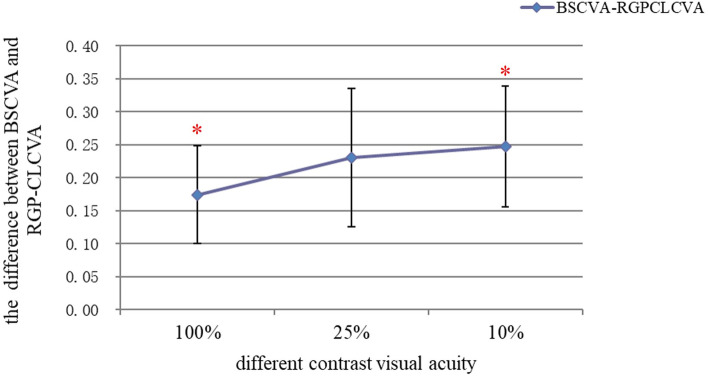
A linear chart of the difference of the corrected vision between BSCVA and RGP-CL-corrected visual acuity at different contrast levels. Bars denote ± 1 standard error of the mean (SEM). * Significant differences (*P* = 0.036)

The differences between BSCVA and RGP-CL-corrected visual acuity at the 100%, 25%, and 10% contrast levels were 0.174 ± 0.074 logMAR, 0.230 ± 0.105 logMAR, and 0.247 ± 0.091 logMAR, respectively. With the decrease in the contrast level, RGP-CL-corrected visual acuity increased more significantly than BSCVA, and the increase at the 10% contrast was significantly better than that at the 100% contrast (*P* = 0.036) (shown in Fig. 2).

### Comparison of the losses of UCVA, BSCVA, and RGP-CL-corrected visual acuity at different contrast levels

The losses of UCVA, BSCVA, and RGP-CL-corrected visual acuity at the 10% contrast and the 25% contrast were significantly different (*P* < 0.05). The losses related to RGP-CLs were smallest. The mean values of the 10% and 25% contrast vision losses are shown in Table 2.

**Table 2:** Comparison of the losses of UCVA, BSCVA, and RGP-CL-corrected visual acuity at different contrast levels

Contrast vision loss	UCVA	BSCVA	RGP-CL-corrected visual acuity	χ^2^	*P*
25%	0.453 ± 0.181	0.328 ± 0.090	0.231 ± 0.099	11.678	0.003
10%	0.593 ± 0.180	0.569 ± 0.138	0.466 ± 0.094	8.162	0.017
Data are presented as mean ± standard deviation. *P* values stand for comparison of different groups. *UCVA * uncorrected visual acuity, *BSCVA* best spectacle corrected visual acuity					

### Comparison of wavefront aberrations in the anterior corneal surface and the anterior surface of RGP-CLs

The results of wavefront aberrations at the anterior corneal surface before and 1 month after RGP-CL wear are summarized in Table 3. The stigmatism Z^− 2^_2_ and Z^2^_2_ and spherical aberration Z^0^_4_ were significantly improved after wearing RGP-CLs as compared to those before the lens wear (*P* < 0.05), while the coma terms Z^− 1^_3_ and Z^1^_3_ were not significantly improved (*P* > 0.05).

**Table 3:** Comparison of wavefront aberrations of the anterior surface of cornea before and after RGP-CL wear

Wavefront aberrations	AstigmatismZ^− 2^_2_	AstigmatismZ^2^_2_	ComaZ^− 1^_3_	ComaZ^1^_3_	SphericalZ^0^_4_
before lens wear	3.761 ± 2.309	3.316 ± 2.147	1.693 ± 1.010	2.195 ± 1.387	3.734 ± 1.061
after lens wear	2.637 ± 1.722	2.016 ± 1.184	1.462 ± 1.068	1.898 ± 1.248	2.622 ± 0.725
t	3.095	2.516	0.697	0.683	3.897
*P*	0.007	0.023	0.496	0.505	0.001
Data are presented as mean ± standard deviation					

## Discussion

After the treatment by keratoplasty, patients often recover corneal transparency successfully, but the visual acuity at the high contrast condition remains low, [19, 20] which may be correlated with corneal wavefront aberrations [21–23]. In this study, a gradual decrease in UCVA was found in post-keratoplasty patients when the contrast was reduced. The improvement of BCVA with spectacles also depended on the contrast levels. When the RGP-CLs were applied to correct the refractive errors, the improvement in visual acuity at all the contrast levels was more significant compared to the use of spectacles, particularly at the lower contrast levels. Therefore, it is necessary to identify the change of wavefront aberrations after RGP-CL wear in post-keratoplasty patients for better improvement of visual performance.

The effect of Zernike aberrations on the optical quality of human eyes varies greatly, with spherical aberration, defocus, astigmatism, and coma in a descending order [24]. The wavefront aberrations have been reported to be reduced with RGP-CL wear in keratoconic patients, including both lower and higher order aberrations, [25, 26] but Romero-Jiménez et al. [27] disclosed that patients with keratoconus can improve high-order aberrations of the anterior corneal surface by wearing RGP-CLs. Greg et al. [28] showed that patients who received keratoplasty had an average reduction of 66% in total higher-order aberrations, 83% in spherical aberration, and 71% in coma by wearing RGP-CLs. In this study, it was found that astigmatism and spherical aberration were significantly reduced with the RGP-CLs for post-keratoplasty patients. Therefore, we believe that the aberration control of the RGP-CLs plays an important role in the improvement of the visual performance for patients after keratoplasty, especially the low contrast viual acuity.

Previous studies have revealed significant irregular astigmatisms in 62.9–77.7% of patients after keratoplasty, and these refractive errors could not be corrected with the spectacles [29, 30]. Uncorrected higher-order aberrations have also been reported to be reduced by wearing RGP-CLs in keratoconic patients, and the contrast sensitivity at the low to middle spatial frequencies can be improved [31, 32]. In our series, the UCVA was worse than 0.3 logMAR for all patients after keratoplasty, and 78.9% of the patients remained the visual acuity of worse than 0.3 logMAR after wearing spectacles. Moreover, anisometropia made it difficult for monocular patients to adapt to spectacles. With the correction by the RGP-CL, 100% of the patients were able to get out of low vision, and 63.2% of the patients obtained corrected vision of above 0.3 logMAR, which was more conducive to achieving simultaneous vision of both eyes [33]. The RGP-CL-corrected visual acuity was better than BSCVA at both high and low contrast levels, especially at the low contrasts. The RGP-CLs may significantly improve patients’ night vision and visual-spatial discrimination, enhancing the quality of life.

The limitations of our study are the small sample size and the use of a single series of postoperative irregular lenses. Besides the Rose-k series, the scleral contact lenses are gradually applied in patients after keratoplasty [34, 35]. A larger cohort of patients with more kinds of contact lenses are needed for further investigation.

## Conclusion

In conclusion, RGP-CL wear effectively improves visual performance of post-keratoplasty patients, particularly visual acuity at lower contrast levels. The reduction of corneal aberrations, mainly the spherical and astigmatism aberrations, with RGP-CLs contributes to the visual improvement at varying contrast conditions.

## Data Availability

The data analyzed during the current study are available from the corresponding author on reasonable request.

## Abbreviations

RGP-CL: rigid gas permeable contact lens
PK: penetrating keratoplasty
UCVA: uncorrected visual acuity
BSCVA: best spectacle corrected visual acuity
CVA: contrast visual acuity
LCL: low contrast loss

## References

[1] Das AV, Basu S (2021). Indications and prognosis for keratoplasty in eyes with severe visual impairment and blindness due to corneal disease in India. Br J Ophthalmol.

[2] Bonneau S, Tong CM, Yang Y, Harissi-Dagher M (2022). The treatment of end-stage corneal disease: penetrating keratoplasty compared with Boston type 1 keratoprosthesis. Graefes Arch Clin Exp Ophthalmol.

[3] Gao H, Liu M, Li N, Chen T, Qi X, Xie L, Shi W (2022). Femtosecond laser-assisted minimally invasive lamellar keratoplasty for the treatment of advanced keratoconus. Clin Exp Ophthalmol.

[4] Gogri P, Bhombal FA (2020). A new technique for fitting of tricurve rigid gas-permeable contact lens in penetrating keratoplasty eyes using Scheimpflug imaging. Indian J Ophthalmol.

[5] Zhao F, Zhao G, Weijie F, Chen L (2018). Application of 3D printing technology in RGPCL simulation fitting. Med Hypotheses.

[6] Kumar M, Shetty R, Lalgudi VG, Roy AS, Khamar P, Vincent SJ (2022). Corneal biomechanics and intraocular pressure following scleral Lens wear in penetrating Keratoplasty and Keratoconus. Eye Contact Lens.

[7] Ozkurt Y, Atakan M, Gencaga T, Akkaya S (2012). Contact lens visual rehabilitation in keratoconus and corneal keratoplasty. J Ophthalmol.

[8] Gruenauer-Kloevekorn C, Kloevekorn-Fischer U, Duncker GI (2005). Contact lenses and special back surface design after penetrating keratoplasty to improve contact lens fit and visual outcome. Br J Ophthalmol.

[9] Feizi S, Zare M (2011). Current approaches for management of postpenetrating keratoplasty astigmatism. J Ophthalmol.

[10] Fares U, Sarhan AR, Dua HS (2012). Management of post-keratoplasty astigmatism. J Cataract Refract Surg.

[11] McLeod SD (2001). Beyond snellen acuity: the assessment of visual function after refractive surgery. Arch Ophthalmol.

[12] Applegate RA, Hilmantel G, Howland HC, Tu EY, Starck T, Zayac EJ (2000). Corneal first surface optical aberrations and visual performance. J Refract Surg.

[13] Shi YH, Wang LY, Lü TB, Qin J (2011). [Changes of ocular higher order aberration in keratoconus eyes wearing rigid gas-permeable contact lens]. Zhonghua Yan Ke Za Zhi.

[14] Jinabhai A, Radhakrishnan H, O’Donnell C (2010). Visual acuity and ocular aberrations with different rigid gas permeable lens fittings in keratoconus. Eye Contact Lens.

[15] Hashemi H, Pakzad R, Heydarian S, Yekta A, Ostadimoghaddam H, Mortazavi M, Ramin S, Khabazkhoob M (2020). Keratoconus Indices and their determinants in healthy eyes of a Rural Population. J Curr Ophthalmol.

[16] Wang Y, Zhao K, Yang X, He J, Wang W (2011). Higher order aberrations and low contrast vision function in myopic eyes (-3.00 to -6.00 d) under mesopic conditions. J Refract Surg.

[17] Bullimore MA, Olson MD, Maloney RK (1999). Visual performance after photorefractive keratectomy with a 6-mm ablation zone. Am J Ophthalmol.

[18] Pesudovs K, Schoneveld P, Seto RJ, Coster DJ (2004). Contrast and glare testing in keratoconus and after penetrating keratoplasty. Br J Ophthalmol.

[19] Zadnik K, Barr JT, Edrington TB, Nichols JJ, Wilson BS, Siegmund K, Gordon MO (2000). Corneal scarring and vision in keratoconus: a baseline report from the collaborative longitudinal evaluation of Keratoconus (CLEK) Study. Cornea.

[20] Betts AM, Mitchell GL, Zadnik K (2002). Visual performance and comfort with the Rose K lens for keratoconus. Optom Vis Sci.

[21] Muftuoglu O, Prasher P, Bowman RW, McCulley JP, Mootha VV (2010). Corneal higher-order aberrations after Descemet’s stripping automated endothelial keratoplasty. Ophthalmology.

[22] Feizi S, Javadi MA, Mohammad-Rabei H (2016). An analysis of factors influencing quality of Vision after big-bubble Deep Anterior Lamellar Keratoplasty in Keratoconus. Am J Ophthalmol.

[23] Yamaguchi T, Satake Y, Dogru M, Ohnuma K, Negishi K, Shimazaki J (2015). Visual function and higher-order aberrations in eyes after corneal transplantation: how to improve postoperative quality of Vision. Cornea.

[24] Oliveira CM, Ferreira A, Franco S (2012). Wavefront analysis and Zernike polynomial decomposition for evaluation of corneal optical quality. J Cataract Refract Surg.

[25] Jinabhai A, Radhakrishnan H, Tromans C, O’Donnell C (2012). Visual performance and optical quality with soft lenses in keratoconus patients. Ophthalmic Physiol Opt.

[26] Yang B, Liang B, Liu L, Liao M, Li Q, Dai Y, Zhao H, Zhang Y, Zhou Y (2014). Contrast sensitivity function after correcting residual wavefront aberrations during RGP lens wear. Optom Vis Sci.

[27] Romero-Jiménez M, Santodomingo-Rubido J, Flores-Rodríguez P, González-Méijome JM (2015). Short-term corneal changes with gas-permeable contact lens wear in keratoconus subjects: a comparison of two fitting approaches. J Optom.

[28] Gemoules G, Morris KM (2007). Rigid gas-permeable contact lenses and severe higher-order aberrations in postsurgical corneas. Eye Contact Lens.

[29] Rocha GA, Miziara PO, Castro AC, Rocha AA (2017). Visual rehabilitation using mini-scleral contact lenses after penetrating keratoplasty. Arq Bras Oftalmol.

[30] Wietharn BE, Driebe WT (2004). Fitting contact lenses for visual rehabilitation after penetrating keratoplasty. Eye Contact Lens.

[31] Negishi K, Kumanomido T, Utsumi Y, Tsubota K (2007). Effect of higher-order aberrations on visual function in keratoconic eyes with a rigid gas permeable contact lens. Am J Ophthalmol.

[32] Marsack JD, Parker KE, Pesudovs K, Donnelly WJ, Applegate RA (2007). Uncorrected wavefront error and visual performance during RGP wear in keratoconus. Optom Vis Sci.

[33] Bandela PK, Satgunam P, Garg P, Bharadwaj SR (2016). Corneal transplantation in Disease affecting only one Eye: does it make a difference to Habitual Binocular viewing?. PLoS ONE.

[34] Barnett M, Lien V, Li JY, Durbin-Johnson B, Mannis MJ (2016). Use of scleral lenses and miniscleral lenses after penetrating Keratoplasty. Eye Contact Lens.

[35] Alipour F, Behrouz MJ, Samet B (2015). Mini-scleral lenses in the visual rehabilitation of patients after penetrating keratoplasty and deep lamellar anterior keratoplasty. Cont Lens Anterior Eye.

